# Congenital Diaphragmatic Hernia

**DOI:** 10.1186/1750-1172-7-1

**Published:** 2012-01-03

**Authors:** Juan A Tovar

**Affiliations:** 1 Professor of Pediatrics, Universidad Autonoma de Madrid, Director, Department of Pediatric Surgery, Hospital Universitario La Paz, Madrid, Spain

**Keywords:** Congenital, Diaphragm, Hernia, Retinoids, Lung, Hypoplasia, Pulmonary, Hypertension, Surgery, Fetoscopy

## Abstract

Congenital Diaphragmatic Hernia (CDH) is defined by the presence of an orifice in the diaphragm, more often left and posterolateral that permits the herniation of abdominal contents into the thorax. The lungs are hypoplastic and have abnormal vessels that cause respiratory insufficiency and persistent pulmonary hypertension with high mortality. About one third of cases have cardiovascular malformations and lesser proportions have skeletal, neural, genitourinary, gastrointestinal or other defects. CDH can be a component of Pallister-Killian, Fryns, Ghersoni-Baruch, WAGR, Denys-Drash, Brachman-De Lange, Donnai-Barrow or Wolf-Hirschhorn syndromes. Some chromosomal anomalies involve CDH as well. The incidence is < 5 in 10,000 live-births. The etiology is unknown although clinical, genetic and experimental evidence points to disturbances in the retinoid-signaling pathway during organogenesis. Antenatal diagnosis is often made and this allows prenatal management (open correction of the hernia in the past and reversible fetoscopic tracheal obstruction nowadays) that may be indicated in cases with severe lung hypoplasia and grim prognosis. Treatment after birth requires all the refinements of critical care including extracorporeal membrane oxygenation prior to surgical correction. The best hospital series report 80% survival but it remains around 50% in population-based studies. Chronic respiratory tract disease, neurodevelopmental problems, neurosensorial hearing loss and gastroesophageal reflux are common problems in survivors. Much more research on several aspects of this severe condition is warranted.

## Disease name/synonyms

Congenital Diaphragmatic Hernia (CDH), ORPHA2140, OMIM 142340, 610187, 306950 and 222400

### Definition

**Figure 1:**
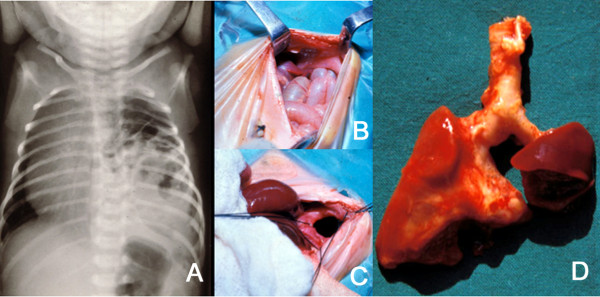
**A: Plain X-ray of the thorax of a newborn with CDH**. There are bowel loops into the left hemi-thorax, the mediastinum is displaced to the contralateral side and the space occupied by the lung is reduced. B and C: At laparotomy, a left, posterolateral diaphragmatic hernia was discovered. In B, small bowel loops can be seen entering the thorax through the orifice. In C, this is seen after reducing the contents of the hernia. D: The patient died of severe persistent pulmonary hypertension days later. At autopsy, extreme left lung hypoplasia and less severe right lung hypoplasia were discovered.

CDH consists of a posterolateral defect of the diaphragm, generally located on the left side, that allows passage of the abdominal viscera into the thorax. The mediastinum is displaced to the contralateral side, the lungs are hypoplastic (Figure 1) and their arterioles are abnormal causing pulmonary hypertension. Respiratory and cardiovascular functions are severely compromised at birth and this, together with the frequently associated malformations, cause considerable mortality and morbidity. CDH was described many years ago [1,2] but survival after repair was not achieved until the 20^th ^century. Pioneers of pediatric surgery [3] reported amazingly low mortalities until the actual severity of the condition surfaced when abortions, stillbirths and pre-hospital deaths were considered, adding a "hidden mortality" to operative and postoperative demises [4]. The pathophysiology of lung insufficiency and persistent pulmonary hypertension that threaten survival are currently better understood, but the results remain disappointing since mortalities near 50% are still reported when all deaths are taken into account in population-based series [5]. CDH management indeed remains one of the major challenges of perinatal medicine and surgery and active research on its mechanisms is warranted.

### Epidemiology

CDH is a rare condition that occurs in < 1-5:10000 births [6]. It seems to be slightly more frequent in males and less frequent in blacks [7,8].

### Clinical features

CDH can be detected during fetal life when screening ultrasonography demonstrates herniation of the intestine and/or the liver into the thorax. Polyhydramnios may lead to antenatal diagnosis in some severe cases [9].

Neonatal symptoms of CDH are heralded by respiratory distress with insufficient oxygenation, excavated abdomen with sternal protrusion and displacement of the heart sounds to the contralateral side. In severe cases, APGAR scores at 1 and 5 minutes are low [10,11]. Respiratory bruits are absent or decreased on the affected side. Unless energetic treatment is undertaken, respiratory condition deteriorates rapidly until the patient dies. The symptoms of insufficient gas exchange are associated with those of persistent pulmonary hypertension [12,13] caused by arteriolar constriction and closure of the pulmonary arterial bed that forces maintenance of a pattern of persistent fetal circulation in which the blood from the right ventricle is shunted to the left heart preventing effective gas exchange. In some cases, this pulmonary hypertension intervenes after some hours during which adaptation to a post-natal circulatory pattern with patent pulmonary circulation had taken place. Hypoxia, acidosis, stress or other causes may bring this "honeymoon" period to an end and re-establish the fetal pattern [14].

In some cases without neonatal symptoms, CDH may manifest itself at any age by mild respiratory distress or it can even be an unexpected finding during a medical check-up for other reasons [15]. In these cases, a hernial sac is more often present [16].

Other organs may be involved in CDH [17] because associated malformations are frequent [18]. The heart and great vessels are often abnormal in CDH patients. Cardiovascular defects like peri-membranous ventricular septal defect, cardiac outflow anomalies (tetralogy of Fallot, double outlet right ventricle, transposition of the great vessels and others) and abnormal great vessels (right aortic arch, double aortic arch, truncus arteriosus, abnormal subclavian arteries and others) are found in about one third of CDH patients [19,20]. Heart hypoplasia, particularly of the left side [21], has also been described [22] but its participation in the clinical picture is still unclear.

Musculoskeletal defects like anomalies of the limbs or of the number and shape of the vertebral bodies and/or ribs [18,23-25], neural tube defects [18,26], abdominal wall defects [27], craniofacial defects [28] or urinary tract anomalies [29] are also found. The parafollicular C-cells [30] and enteric innervation are deficient and might account for some dysfunctions [31]. Finally, the presence of the intestine in the thorax during late fetal development causes malrotation and/or malfixation [32] that can further complicate the disease [33].

### Etiology

The causes of CDH are largely unknown. Most cases are isolated, but associated malformations are often observed [34], sometimes as components of Pallister-Killian and Fryns [35], Ghersoni-Baruch [36], WAGR and Denys-Drash [29,37], Brachman-De Lange [38], Donnai-Barrow [39] or Wolf-Hirschhorn [40] syndromes. CDH is also observed in some chromosomal anomalies whether related or not to these syndromes like 9p tetrasomy [41], 11q23-qter duplication [42], 15q24-26 [43], 15q26 [44], 1q41-q42.12 [39,45] and 8p23.1 [46] deletions. Null-mice for several genes, like shh transcription factors Gli 2 and Gli3 [47], Slit3 [48,49], COUP-TFII [50], Fog2 [51], Wt1 [52] and FGFLR-1 [53] have CDH.

The orifice in the diaphragm is caused by delayed or disturbed separation of the thoracic and abdominal compartments of the body by closure of embryonic pleuroperitoneal canals effected by growth of the post-hepatic mesenchymal plate and of the pleuroperitoneal folds [54-57].

CDH occurs more often on the left side (4:1) [58]. In some rare instances [59], the defect is a true agenesis of the hemidiaphragm [60], but in most cases it is limited to the posterolateral area. Less often, there is a hernial sac devoid of muscular fibers [16]. When the defect is located on the left side, the thorax may contain small and large bowel, the spleen, the stomach, the left lobe of the liver and, occasionally, the kidney. Right-sided CDHs usually contain part of the right lobe of the liver and sometimes the bowel and/or the kidney.

The lung is obviously hypoplastic on the side of the hernia, but the contralateral one is also affected to a variable extent. Lung weight is decreased and the number of alveoli is reduced due to insufficient branching [61]. Respiratory epithelial maturity is delayed with hyaline membrane disease patterns similar to those found in prematures [62]. Surfactant deficiency has been shown [63-65] although this issue is controversial [66]. The distal bronchiolar arteries have muscularized walls and the wall thickness is increased particularly at the expense of the media and adventitial layers [67,68].

The lung hypoplasia, immaturity and arteriolar thickening that accompany human CDH can be due to prenatal compression of the lung by the herniated viscera. All these lesions can be reproduced by surgically creating a CDH in rabbits [69-71] lambs [72-74] and primates [75]. These models were used for investigating various aspects of CDH and also for developing fetal surgery [76-81]. However, the associated malformations are absent in these mechanical or surgical models and this limits to some extent their validity as research tools. In fact, the assumption that lung hypoplasia is purely mechanical is probably incorrect because it is already present in teratogenic models of CDH before any herniation has taken place. A "dual hit" pathogenesis [82], in which abnormal development followed by compression cause lung hypoplasia is widely accepted. Regardless of the origin of the lung lesions, these consist of insufficient airway branching [61,63,83], reduced gas exchange surface, abnormal sacculo-alveolar maturation [64,84] and abnormal, muscularized distal arterioles [68,85].

**Figure 2:**
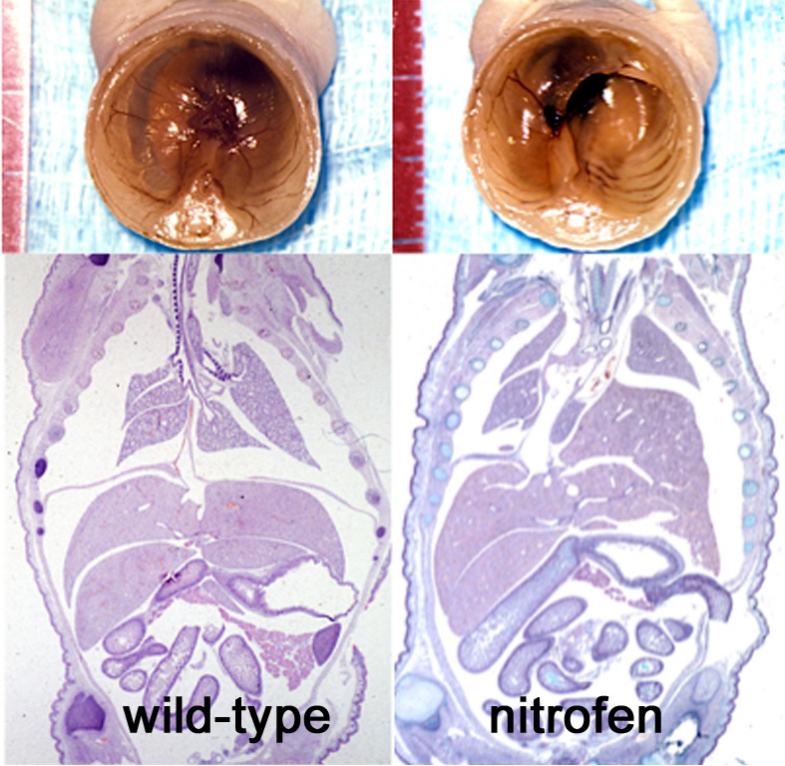
**Experimental CDH in rats**. Administration of 100 mg of the herbicide nitrofen on gestational day 9.5 to pregnant rats induced the malformation in 40 to 80% of the offspring. On the left, neonatal autopsy of an untreated, wild-type rat shows an intact diaphragm (above) that can also be seen in the frontal section of the trunk (below). On the right, similar preparations of a rat with nitrofen-induced CDH show a large postero-lateral diaphragmatic orifice, herniation of the liver into the thorax, displacement of the mediastinum to the contralateral side and lung hypoplasia.

Pharmacologic or teratogenic models of CDH have been crucial for unveiling some pathogenic mechanisms. Prenatal administration of oxidant chemicals like the herbicide nitrofen (2,4-dichloro-phenyl-p-nitrophenyl ether), 4-biphenyl carboxylic acid, bisdiamine, and SB-210661, induce CDH in rodent fetuses. These chemicals inhibit in vitro retinol-dehydrogenase-2 (RALDH-2) a key enzyme for the production of retinoic acid (RA) [86]. Among these agents, nitrofen has been the more extensively used [54-56,87-97] because of the striking resemblance of the diaphragmatic, pulmonary and other associated defects to those of the human condition (Figure 2). Proliferation of the pleuroperitoneal folds is arrested in rats with CDH [98] and the timing of this arrest probably conditions the laterality of the defect. Fibroblast growth factor (FGF) receptor-like 1 (FGFRL1) [99] and WT1 [97] are downregulated in these diaphragms.

Although the mechanism by which CDH is induced in these models is not well known, disturbances of the retinoid signalling pathway, a key regulator of embryonic morphogenesis, are likely [90]. Vitamin A deficient-rats give birth to pups with CDH [100]. RA metabolism perturbations caused by nitrofen can be alleviated by addition of either vitamin A or RA [95,101-103]. Hypoplastic lungs from rats with nitrofen-induced CDH show decreased RA synthesis [104] and deficient retinol transportation [105], whereas RA rescues lung hypoplasia [93] probably by upregulating COUP-TFII, FOG2, and GATA4 [106] that are known to be necessary for both pulmonary and diaphragmatic development [39,50]. On the other hand, compound knock-out mice for retinoic acid receptors (RAR) have phenotypes involving CDH and several of the associated malformations seen in this condition [107] whereas blockade of RAR with BMS493 induces CDH [108] and administration of BMS-189453, a RA antagonist, to pregnant mice reproduces the phenotype of CDH [109].

A similar involvement of the retinoid signaling pathway is likely in human CDH because retinol and retinol-binding protein were decreased in the blood of a group of newborns with this malformation [110]. This has recently been confirmed in a larger case-control study [111]. Moreover, some of the genes involved in the pathogenesis of human CDH are tightly related to retinoid signaling [112-115].

Another factor that might contribute to lung hypoplasia in CDH is decreased airway pressure during fetal life. Fetuses with tracheal atresia have large lungs [116,117] whereas fetal tracheostomy induces lung hypoplasia [118]. It has been shown that airway peristalsis is abnormal in lung explants from rat embryos treated with nitrofen and it is likely that decreased airway pressure could contribute to hypoplasia [92,119].

### Diagnosis

As mentioned before, CDH is often ultrasonographically diagnosed before birth [120,121]. The intestine and/or the liver may be in the thorax and the lungs are small. US scan allows detailed assessment of the heart. Lung growth is measured as a proportion of head growth. This lung-to-head ratio (LHR) has some prognostic value [122-127] because when it is below 1, survival is compromised [125,128,129]. However, the accuracy of these measurements is questionable [130] and other alternatives like lung/thorax (L/T) transverse area ratio [126,131] or volumetry by MRI have been developed [132-136]. The observed-to-expected LHR (o/e LHR) seems to be a reliable predictor of severity (patch requirement, ECMO) and survival [127,137]. The position of the liver is also of unquestionable value, since liver-up cases more often require ECMO support and have worse survival [138-140]. The intra-thoracic position of the stomach has less value for this purpose [141,142]. When diagnosis is made *in utero*, amniocentesis is often performed for detecting chromosomal aberrations [143] and may help to estimate lung maturity [64].

After birth, the diagnosis is readily made on the basis of symptoms and physical signs. A plain X-ray of the thorax and abdomen informs of the position of the herniated viscera. Blood gases and pH status reflect the efficiency of gas exchange and other derived indexes refine this assessment [144,145]. Ultrasonography of the heart is necessary for ruling-out associated malformations, for measuring the right-to-left shunt and for estimating the severity of pulmonary hypertension [146-148]. Measurements of the pulmonary artery diameters and the use of some indexes derived from them may facilitate this task [149-151]. Cardiac U.S. is one of the more reliable methods for determining when the patient is "stabilized".

### Differential diagnosis

It is rarely necessary to rule out other conditions because CDH is often detected before birth and because diagnosis is easy. Physical exam may suffice but passing a naso-gastric catheter into the stomach before a plain X-ray of the thorax and abdomen may help to locate it or to detect esophageal displacement. In some rare instances, X-rays may suggest a cystic malformation of the lung but again the position of the stomach and the contour of the intra-abdominal gas bubbles facilitate distinction of both conditions.

### Treatment before birth

Since the introduction of routine prenatal U.S. screening, a large proportion of fetuses with CDH are diagnosed *in utero*. Termination of gestation is sometimes preferred, particularly when chromosomal aberrations and syndromes are present [5,152-154]. The possibility of fetal instrumentation directed to alleviate the consequences of the herniation is becoming a progressively more acceptable alternative. Thirty years ago Harrison and coll. started pioneer work in San Francisco aimed at developing animal models of CDH on which to test the effects of prenatal manipulation. After demonstrating in fetal lambs that lung compression led to lung hypoplasia [73] and that prenatal decompression reversed this condition [72], this group undertook the surgical creation of CDH [74] and later on, the demonstration of reversal of lung hypoplasia and arteriolar thickening when the diaphragmatic defect was repaired before birth [155]. Clinical application of this rationale led, with close consideration of all the ethical and technical issues involved, to the first attempts at prenatal repair of CDH in human fetuses [120,156,157]. The position of the fetal liver [138] and the demonstration of the lethal effect of umbilical vein compression during fetal reduction of the left lobe of the liver into the abdomen during surgery [74], limited considerably the number of cases suitable for this approach. Furthermore, a randomized study demonstrated that fetal repair did not produce better outcomes than optimal postnatal treatment [158]. The trial was discontinued and a new approach, based on the observation of lung hyperplasia in congenital tracheal obstruction, was developed. The evidence that airway pressure had something to do with lung development led to assume that tracheal occlusion during fetal life could counteract the effects of lung compression in fetuses with CDH. This was tested in the fetal lamb [77,78,159-161] and then in the human fetus [162,163]. Another randomized trial was discontinued when a significant shortening of gestation was observed in fetuses treated in this manner [164]. However, the effects of tracheal occlusion were indeed very positive in terms of alveolar size and histology [159]. Maturation and surfactant function were apparently not so much improved, unless the plug was reversed before delivery [165].

The concurrent development of minimally invasive surgery had the immediate effect of making fetendoscopic balloon tracheal occlusion possible [75,163]. Considerable experience rapidly accumulated and randomized trials are currently underway concentrating these efforts in fetuses with LHR below 1 considered otherwise unviable [166]. Survivals approaching 50% in this group are encouraging [139] and much information has already accumulated on the structure, maturity and tracheal pathology of survivors.

Experimental and clinical evidence suggest that biochemical lung maturation is delayed in fetuses with CDH [65,167-169] and this led to the proposal that this process be accelerated by administration of maternal corticosteroids as it is done for premature deliveries. The evidence of the benefits of this medication is not totally convincing [170,171] and therefore, a multicenter trial could be necessary [172].

### Treatment after birth

Whenever prenatal diagnosis is made, it is advisable to direct the mother to a tertiary perinatal center in which all the necessary obstetric, neonatal and surgical skills are concentrated [173]. Gestation should be prolonged until near term if possible [174] (this can be hard to achieve in cases with polyhydramnios) and, although it is often preferred [175], there is no evidence of the benefits of delivery by Cesarean section [174,176,177].

When CDH becomes symptomatic after birth and in all cases diagnosed prenatally, a careful protocol of respiratory assistance should be implemented. Immediate postnatal intubation avoiding mask ventilation should be carried out, and small-volume, high frequency and reduced peak pressure mechanical ventilation should be started. Oxygen delivery has to be tailored for maintaining pre-established gasometric goals. Surfactant has been used in an attempt to compensate for biochemical immaturity in CDH babies [178,179], but this has not been beneficial [180,181]. The stomach is emptied through a nasogastric tube and vascular accesses (peripheral and central veins, umbilical artery) are secured for infusion of fluids, drug administration and blood sampling. A bladder catheter is inserted and urine output is monitored. Then, a period of assessment of the chances of adequate gas exchange is started adapting all actions to the clinical findings. If arterial oxygenation is maintained above a preset minimum, the lung will probably be capable of adequate gas exchange. Pre-ductal and post-ductal percutaneous oxygen saturation measurements help in the assessment of both the adequacy of the lungs to sustain life and of the magnitude of right-to-left shunting. Inotropic drugs are used as necessary and cardiac function and heart anomalies are carefully evaluated. Persistent pulmonary hypertension may cause right ventricle dysfunction and in these cases, maintenance of the patency of the ductus arteriosus is indicated [182].

After years of frustrating results of application of respiratory assistance goals usually applied to other conditions, it was realized that the hypoplastic and probably immature lung of CDH was severely damaged by excessive oxygen delivery and, particularly by excessive airway pressure that led to pneumothorax, barotrauma and volutrauma [183,184]. Mortality decreased drastically and all these untoward consequences of ventilator assistance became milder when a policy of "gentle ventilation" was implemented [185]. Spontaneous ventilation when possible or high frequency, low pressure ventilation (< 20-25 cm H_2_O), no relaxation, alkalinisation and adoption of modest gasometric goals (pre-ductal saturation of 80-95%, PaO_2 _around 60 mmHg and "permissive" hypercapnia of up to 60 mmHg) drastically changed the results [186]. This policy progressively gained adepts and it is nowadays the standard in most developed countries [187-189].

High-frequency, oscillatory ventilation is also widely used in these patients [190-192] in whom it allows adequate oxygenation and CO_2 _elimination with very low airway pressures [193]. Lung damage is apparently minimized while attaining the modest gasometric objectives set. A multicenter European trial is currently underway [194].

For how long time these treatments are necessary varies widely from one patient to another depending on the degree of lung hypoplasia, on the arteriolar reactivity and therefore on the duration of persistent pulmonary hypertension and on the associated malformations, particularly the cardiovascular ones.

The treatment of pulmonary hypertension is of particular concern because it determines shunting of non-oxygenated blood out of the lungs. Several medications have been used: tolazoline [67,195,196] and prostacyclins [197,198] were tried first but they did not produce good enough results. Prostaglandin 1E is occasionally used [199]. Inhaled nitric oxide, a well known smooth muscle relaxant is, in turn, widely used [200-203] with variable results although there is no strong evidence of its benefits [204]. Inhibitors of phospho-diesterase like sildenafil, known for their vasodilator action, are currently used in some cases [205] but, again, there is only anecdotal evidence of their benefits. These medications are coupled with inotropic agents, like dobutamine (up to 20 mg/kg/min), dopamine (< 10 mg/kg/min) [206] and also, sometimes with peripheral vasoconstrictors, like adrenaline at low doses, aimed at reducing the shunting by increasing pressure in the systemic circulation.

### ECMO

Extra-corporeal membrane oxygenation (ECMO) may be a useful adjunct in the treatment of CDH. Cannulation of both the right carotid artery and jugular vein and connection to a circuit with a membrane gas exchange chamber allows oxygenation and CO_2 _disposal without participation of the lung which is preserved from any pressure insult [207]. Alternatively, veno-venous ECMO avoids cannulation of the carotid artery while permitting adequate gas exchange [208-210].

ECMO can be seen as a safety net maintained until proper gas exchange is demonstrated at weaning test periods. The fact that institutions, particularly in the US, which used ECMO liberally [10] had results comparable to Canadian institutions [211] that rarely, if ever, used it, casted some doubt on the actual need for this complicated and risky procedure [12,212]. The criteria for ECMO indication are variable, but they can be summarized as follows: Inability to reach pre-ductal SatO_2 _> 85% or post-ductal SatO_2 _> 70%, oxygenation index (mean airway pressure × FiO_2 _× 100/PaO_2_) ≥ 40 or need for high peak inspiratory pressures or a-AdO_2 _greater than 600 for 6 to 8 hours [213]. The main contraindication is, of course, the inability to obtain SatO_2 _> 80% at any moment of the initial treatment under FiO_2 _of 1. In fact, ECMO registries showed that the worst results were obtained precisely in the CDH group of patients [214,215]. This, together with the limitations of the technique (required weight above 2000 g, need for heparinisation), somewhat tempered the initial enthusiasm and the proportion of patients so treated decreased everywhere to more reasonable figures. There is only weak evidence of the benefits of ECMO in this particular group of patients [216] because only non-randomized trials have been carried out on them [171,212] and the only RCT reported in the UK was not specifically directed to babies with CDH [217]. This technique is probably a good adjunct in a limited number of patients in which predicted severe lung hypoplasia would make adequate gas exchange impossible or in those in whom reversal to the fetal pattern of circulation becomes unmanageable.

### Surgical repair

Surgical repair of CDH used to be in the past a life-saving emergency. It is presently accepted that it should be undertaken only after cardio-respiratory functions are stable. A policy of "delayed" surgery coupled with gentle ventilation and occasionally ECMO support yields the best results recorded. For how long surgery has to be delayed is unclear, but a few days and even weeks may be beneficial. The goal of all preoperative treatments is to obtain "stabilization" of the patient and this means acceptable oxygenation (PaO_2 _> 40 mmHg) and CO_2 _disposal (PaCO_2 _< 60 mmHg) with stable pulmonary pressures (< 50% of systemic pressure), tolerable shunting, good myocardial function and adequate renal clearance with reduced or withdrawn inotropic drugs. Obviously, there are no magic indicators of stabilization (this may also be impossible) and a decision to operate is only made after a consensus among all the actors of these treatments has been reached. Once again, when critically reviewed, even the alleged benefits of delayed repair are not consistently documented [218].

In many institutions, the operation is carried out in the neonatal ICU in order to minimize changes that might increase stress [219-221]. Under general anesthesia, a subcostal or transverse abdominal incision is made, the herniated viscera are carefully reduced into the abdomen and the diaphragmatic orifice is closed with interrupted sutures without leaving an intercostal tube. A tube was routinely used in the past [58,222] until it was realized that underwater seals cause increased respiratory work and overdistension of the hypoplastic lung that may further reduce ventilation. A tubeless policy was then advised [185,223]. When the defect is too large, a prosthetic patch is used to achieve closure [224,225]. It is sutured to the rims of the orifice with interrupted sutures and, to avoid excessive tension and enlargement of the hemi-thorax, cone-shaping of the patch can be beneficial [226]. The use of a patch seems to increase the risk of re-herniation [227-230] although it should be acknowledged that patients requiring a patch have larger defects which entail higher morbidity [59]. Abdominal wall or latissimus dorsi muscle flaps have also been used for CDH repair [231-234].

Malrotation or non-rotation is usually present but this is rarely a problem in the newborn and the main concern of the surgeon after repair of the hernia is abdominal wall closure without excessive pressure [235,236]. In some cases, another abdominal wall prosthetic patch is necessary to avoid abdominal compartment syndrome [237-239].

Since the advent of minimally invasive surgery (MIS), thoracoscopic [240-246] or laparoscopic [247] approaches have been proposed for CDH repair. Both are indeed possible (with or without patch insertion), although it should never be disregarded that this repair comes only after stable conditions have been secured. Probably, MIS is a good approach in cases diagnosed during infancy or in outborns with less severe symptoms. Nevertheless, some concerns about this particular approach in the newborn period have been expressed on the basis of excessive perioperative hypercapnia and prolonged postoperative low brain oxygenation [248]. The risk of recurrence after a minimally invasive approach has been found to be similar to that of open surgery by some reports [246] but it was indeed increased in other series [248,249].

### Prognosis

The main outcome endpoint is, of course, survival and the wide range of figures reported is puzzling. When only hospital, postoperative results were reported, survival approached 70% [250] or more [189,251,252]. However, those institutions with attached maternity facilities that treated mostly inborns, reported higher mortalities. The introduction of ECMO, in parallel with advances in other aspects of treatment, improved the results and the top institutions reported survivals approaching 90% although, sometimes, their statistics excluded chromosomal aberrations, multiple malformations and even some patients that did not reach surgery. The truth is that still nowadays, if terminations of pregnancy, spontaneous abortions, stillborns, pre-hospital and/or preoperative deaths and surgical mortality are taken into account, real mortality is still between 50% and 60%. But if only hospital statistics are considered, there has been indeed a clearcut progress in the results. Many institutions and the multi-institutional registries report 70% to 80% survival with some institutions peaking at 90%. Right-sided hernias seem to have a worse prognosis than left-sided ones and may require more ECMO support [253-255].

Other in-hospital complications can occur after CDH repair. Pleural effusions and chylothorax may require nutritional treatment, medication and drainage [256-260].

Chronic respiratory tract disease is frequent in these patients. Lung hypoplasia can be caught up only in part during the first months of life and oxygen toxicity, barotrauma and volutrauma may occur even after gentle ventilation. True broncho-pulmonary dysplasia is relatively frequent in survivors [250,261], and a number of them require oxygen at home for long periods of time [228,251]. Restrictive [262-264] and obstructive [263-265] lung diseases have been reported in CDH survivors many years after operation and diaphragmatic rigidity and thoracic deformities can also play a minor role in chronic lung disease [266-268].

Gastroesophageal reflux is frequent in CDH survivors. The diaphragmatic sling may be malformed or absent and the repair changes the anatomy of the region. In addition, mal-rotation may delay gastric emptying, and the abnormal balance of pressures in the thorax and abdomen in the course of the respiratory cycle facilitates retrograde passage of gastric contents to the esophagus [269]. Finally, there is evidence of abnormal enteric innervation in CDH and it is likely for esophago-gastric peristalsis to be abnormal [96,270]. Reflux can complicate the pre-existing respiratory disease and, for all these reasons, a considerable proportion of these patients [271,272] respond poorly to medical treatment and ultimately require anti-reflux surgery [227,273-276]. Prophylactic fun-doplication during surgical repair has been proposed in patients with CDH [277]

Neurodevelopmental deficits are always possible in these patients in whom brain oxygenation was marginal for long periods of time and particularly when ECMO required major vascular occlusion [228,278-283]. Neurosensorial deafness occurs in a small proportion of children surviving CDH [283-290]. This is progressive [287,291] and it is generally considered to be accounted for by prolonged antibiotic treatments [288]. However, other newborns with similar treatments suffer this deafness more rarely and it should be acknowledged that perhaps CDH patients have a particular sensitivity or a developmental defect of the inner ear.

Other sequelae are possible in this particular group of patients. This, and the frequent associated malformations, require long-term follow-up and permanent support.

### Genetic counseling

Most cases are sporadic but there are some reports of familial clusters that suggest multifactoral [292-296] rather than autosomal recessive [297,298] patterns of inheritance. Genetic aspects of CDH were recently addressed [299-302]. Two thirds of these cases were males and the risk of recurrence in sibs is 2% [294].

### Unresolved questions

CDH remains one of the most difficult problems of perinatology and neonatal surgery. Its mechanisms are beginning to be unveiled, but the severity of the lung and other lesions require the use of the entire armamentarium of sophisticated neonatal care. This, together with the rarity of the condition, make the setting of solid evidence-based protocols of management very difficult. The perspectives of fetal manipulation or even of prophylactic drug treatment remain still at an embryonal stage and, most probably, this condition will remain a hot topic of research in the coming years.

## Conclusions

CDH is a complex condition probably caused by disturbed molecular signaling during organogenesis. The diaphragmatic orifice is invariably accompanied by pulmonary hypoplasia with vascular hyper-reactivity that causes deficient gas exchange and persistent pulmonary hypertension. In addition, other malformations that further complicate the clinical course may be present. All efforts are directed at enhancing antenatal lung growth in prenatally diagnosed cases and at protecting the lung during the intensive care pre and post-operative phases in all cases. Fetoscopic reversible tracheal obstruction seems promising before birth and gentle ventilation with occasional ECMO use yields the best results post-natally. Nevertheless, many aspects of the disease are still unknown and, given that the incidence is relatively high, that the expenses involved in the current treatments are overwhelming and that the sequelae are frequent, more research efforts into causation, prevention and treatment are warranted.

## Competing interests

The author declares that they have no competing interests.
